# Blue-yellow combination enhances perceived motion in Rotating Snakes illusion

**DOI:** 10.1177/20416695241242346

**Published:** 2024-04-02

**Authors:** Maiko Uesaki, Arnab Biswas, Hiroshi Ashida, Gerrit Maus

**Affiliations:** Center for Information and Neural Networks (CiNet), Advanced ICT Research Institute, 365190 National Institute of Information and Communications Technology (NICT), Suita, Osaka, Japan; Graduate School of Frontier Biosciences, 13013 Osaka University, Suita, Osaka, Japan; School of Social Sciences, 54761 Nanyang Technological University, Singapore; School of Social Sciences, 54761 Nanyang Technological University, Singapore; School of Social Sciences, 54761 Nanyang Technological University, Singapore; Department of Psychology, 6851 University of Nevada Reno, Reno, NV, USA; Graduate School of Letters, Kyoto University, Kyoto, Kyoto, Japan

**Keywords:** motion, colour, perception, illusion, vision‌

## Abstract

The Rotating Snakes illusion is a visual illusion where a stationary image elicits a compelling sense of anomalous motion. There have been recurring albeit anecdotal claims that the perception of illusory motion is more salient when the image consists of patterns with the combination of blue and yellow; however, there is limited empirical evidence that supports those claims. In the present study, we aimed to assess whether the Rotating Snakes illusion is more salient in its blue-yellow variation, compared to red-green and greyscale variations when the luminance of corresponding elements within the patterns were equated. Using the cancellation method, we found that the velocity required to establish perceptual stationarity was indeed greater for the stimulus composed of patterns with a blue-yellow combination than the other two variants. Our findings provide, for the first time, empirical evidence that the presence of colour affects the magnitude of illusion in the Rotating Snakes illusion.

Visual illusions have provided unique and valuable opportunities to examine the mechanisms underlying perception. One such illusion is the Rotating Snakes illusion, a variant of the Fraser-Wilcox illusion (Fraser & Wilcox, 1979; Kitaoka, 2017), wherein a static image elicits perception of rotational motion (Kitaoka & Ashida, 2003). The Rotating Snakes illusion is composed of colour- or luminance-defined micropatterns, each consisting of four adjacent areas of different colours (e.g., white, yellow, black, blue) or levels of luminance (white, light grey, black, and dark grey). The order in which those areas are arranged within each micropattern dictates the direction of illusory rotational motion (Kitaoka & Ashida, 2003).

The illusion likely occurs due to the varied latency of neuronal responses to the four levels of luminance (Backus & Oruç, 2005; Conway et al., 2005) leading to illusory motion from high-contrast to low-contrast regions (Faubert & Herbert, 1999; but see Atala-Gérard & Bach, 2017), and to the direction-specific bias in motion detector output arising from the temporary asymmetric filtering of spatially asymmetric images (Fermüller et al., 2010; Murakami et al., 2006) or from the saturating nonlinearity of motion detectors (Bach & Atala-Gérard, 2020). Both accounts assume that local motion signals are generated in V1, which are subsequently integrated to yield coherent global illusory motion at the level of MT+. Evidence from a functional magnetic resonance imaging study by Ashida et al. (2012) supports this notion and suggests that illusory motion is cortically represented similarly to real motion.

It has been reported that the perception of illusory motion in the Rotating Snakes illusion is more salient in the peripheral than central visual field and is strongly correlated with eye movements including eye blinks (Kitaoka & Ashida, 2003; Kuriki et al., 2008; Murakami et al., 2006; Otero-Millan et al., 2012). There have also been consistent, albeit anecdotal, observations that the presence of colour, specifically the combination of blue and yellow, may influence the perception of illusory motion (e.g., Kitaoka, 2014). Yet, empirical evidence is limited to support (or contradict) the observation that colour configuration influences the strength of illusory percept in the Rotating Snakes illusion.

In the present study, we aimed to investigate whether the perceived magnitude of illusory motion in the Rotating Snakes illusion depends on the presence or combination of colours. We employed the cancellation method to quantify the strength of illusion, utilising a temporal two-alternative forced-choice (2AFC) paradigm to measure points of subjective equality, and compared the strength of illusion elicited by three chromatic variations of the Rotating Snakes illusion (blue-yellow [BY], red-green [RG], and greyscale [GREY]; see Methods). It was hypothesised that the magnitude of illusion would be quantifiably greater in the BY variation than in the RG and GREY variations.

## Methods

### Participants

Twenty-five healthy volunteers (7 males and 18 females, mean age: 21 years, age range: 18–31 years) participated in the study in accordance with the ethical standards stated in the Declaration of Helsinki and approval from the Institutional Review Board of Nanyang Technological University. Of the participants, undergraduate students were compensated with course credits. All had normal or corrected-to-normal visual acuity, and normal colour vision as examined using colour blindness test (Ishihara, 1917). Amongst the 25 participants, 10 were left-eye dominant and 15 were right-eye dominant, as assessed using a simplified version of the Miles test (Miles, 1929). Written informed consent was obtained from every participant.

### Apparatus

The stimuli were generated using MATLAB (The Mathworks Inc., Natick, MA, USA) with PsychToolbox 3 (Brainard & Vision, 1997; Pelli, 1997) and were displayed on a 21-inch CRT monitor (1152 × 864 pixels, refresh rate 100 Hz; FD Premium, SUN Microsystems, California, US). The viewing distance was 57 cm, fixed by a chinrest. EyeLink 1000 Plus (SR Research, Ottawa, Canada) was used to track participants’ eye movements, accompanied by an infrared illuminator allowing for eye-tracking in the dark, positioned below the CRT monitor.

### Stimuli

The stimulus was a modified version of the Rotating Snakes illusion (Kitaoka & Ashida, 2003), composed of rings of micropatterns, each of which consisted of four stepwise luminance levels. The normalized intensities of the four luminance levels were 1, 2/3, 0, and 1/3. Each ring consisted of 24 cycles of this waveform. The luminance of each of the four elements was matched to that of Conway et al. (2005): the luminance level of the white patch was 70 cd/m^2^, light grey 40 cd/m^2^, black < 1 cd/m^2^, and dark grey 30 cd/m^2^ as measured with a luminance meter (LS-100, KONICA MINOLTA, Tokyo, Japan). The stimulus was presented against a grey background with the mean luminance of 35 cd/m^2^ in the left visual hemifield. The distance between the centre of the stimulus and the centre of the screen (i.e., fixation point) was 12° visual angle, based on the observation by Hisakata and Murakami (2008) that the perceived illusory motion was the strongest at that eccentricity. The stimulus subtended 3.5° around the stimulus centre (7° in diameter).

There were two stimulus types defined by their configurations, which determined the directions of induced illusory motion: counter-clockwise (CCW) and clockwise (CW). The CCW stimuli each comprised an array of colour/luminance micropatterns in such order that they elicited illusory motion in the CCW direction; while the CW stimuli were each composed of an array of colour/luminance micropatterns arranged in the reversed order and elicited illusory motion in the CW direction.

**Figure 1.**
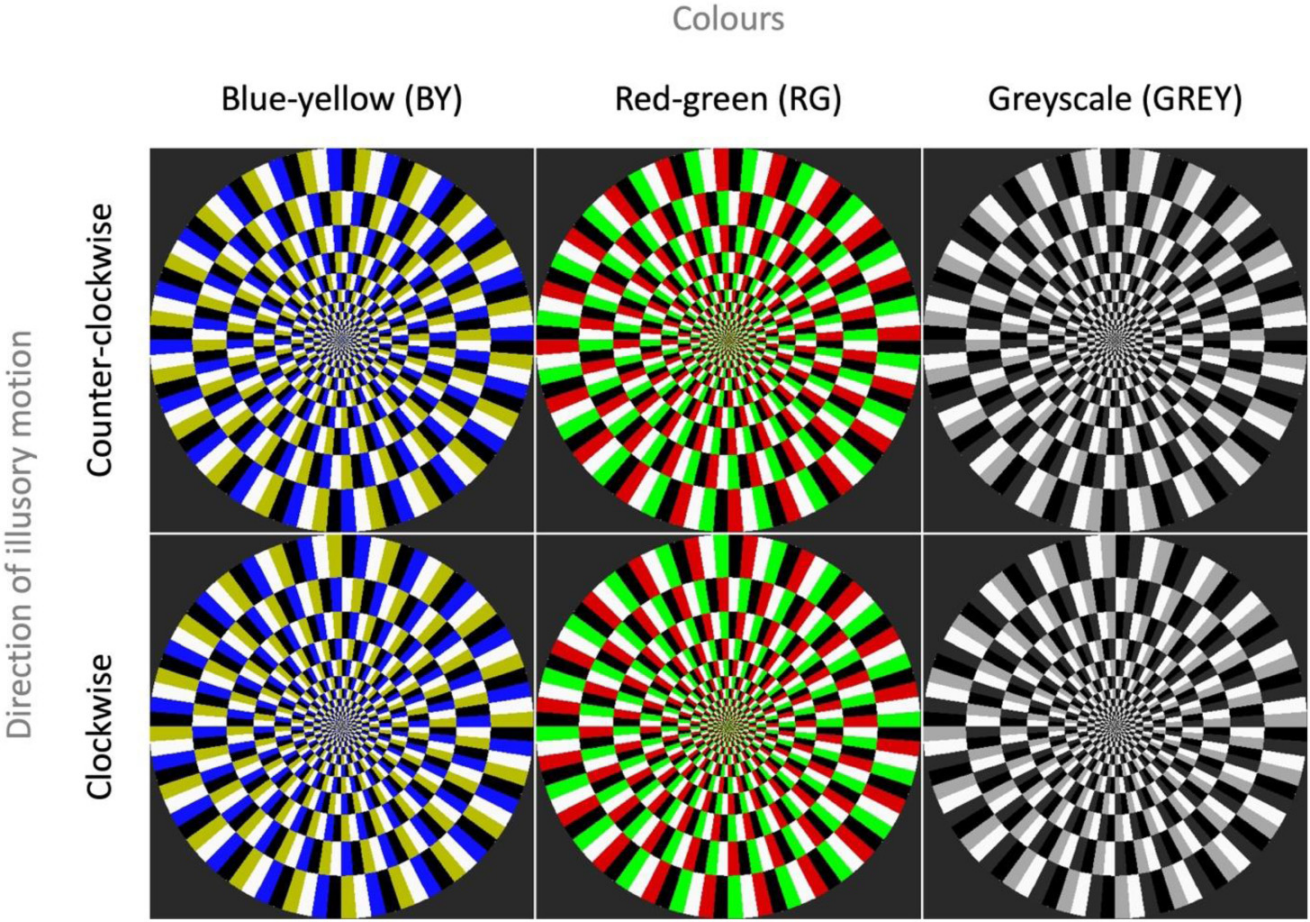
Six stimuli used in the experiment. There were two stimulus types: stimuli in the top row were composed of an array of micropatterns designed to induce perception of counter-clockwise illusory motion (CCW), and those in the bottom row clockwise illusory motion (CW). There were three colour variations for each stimulus type: blue-yellow (BY), red-green (RG), and greyscale (GREY).

For each stimulus type, there were three colour variations: BY, RG, and GREY. The resulting six stimuli are shown in Figure 1. Blue and red elements in BY and RG conditions, respectively, were matched to the dark grey element in GREY condition in luminance; whereas yellow and green elements in BY and RG conditions, respectively, were matched to the light grey element in GREY condition. Coloured elements of the stimuli in BY and RG conditions were originally generated using the following RGB values: [15.3, 16.575, 255] for blue, [183.6, 188.7, 0] for yellow, [216.75, 0, 0] for red, and [0, 255, 0] for green elements. However, since the colour profile of the monitor was modified by loading a colour lookup table, the colours specified above may not necessarily be representative of the colours displayed on the screen. We were unable to measure the colour coordinates of the stimulus elements as they appeared on the monitor with our equipment.

### Procedure

The method of constant stimuli, as in Hisakata and Murakami (2008), was used to measure the velocity of stimulus rotation that yielded the point of subjective stationarity (PSS), at which the illusory motion was cancelled by the physical rotation of the stimulus and the stimulus appeared stationary. The stimulus rotated at velocities ranging from −1.2 to 1.2 deg/s by steps of 0.3 deg/s, where 1 deg/s corresponds to one degree of polar angle per second. Negative sign indicates the CCW direction of physical rotation. The range and step size were determined a priori in a pilot experiment. The stimulus was presented for 500 ms, after which participants made a 2AFC judgment as to whether the stimulus appeared to rotate CW or CCW by pressing either the left or right arrow key on the keyboard. There was no time limit implemented for participants to respond.

Each of the six stimuli (Figure 1) was presented 27 times per block, comprised of three repetitions for each of the nine levels of velocity. The block of resulting 162 trials (3 repetitions × 9 levels of velocity × 6 stimuli) was repeated six times; as a result, a total of 972 responses were collected from each participant. The six blocks were identical in terms of stimuli presented, but the order in which the stimuli were presented and the velocities at which they rotated were randomised within each block.

Participants were asked to sit in the dark for 5 min before the start of the experiment. Participants had their left eye occluded and used the right eye to view the stimulus in a dark room. Participants were instructed to fixate at a cross presented in the centre of the screen during the trials, and the movements of their right eye were tracked throughout each block. At the end of each block, they were allowed to take a break as long as they needed.

### Analysis

Data were analysed using MATLAB and JASP (https://jasp-stats.org/). Participants’ responses were coded as either “0” or “1” and were analysed individually by fitting logistic psychometric functions for each condition and stimulus type. PSS was defined as the velocity of physical rotation at which both CW and CCW responses had an equal probability of occurring. The sign of PSS was then normalised as to the stimulus direction so that positive PSS represents cancelling motion in the anti-stimulus direction that is consistent with expected illusory motion. Subsequently, a two-way analysis of variance (ANOVA) was performed to assess the effects of stimulus type and colour.

## Results

Of the 25 participants, five were excluded from further analysis as their fixation was unstable during more than 50% of all trials, based on the eye-tracking data. Specifically, the average distance between the gaze position and the fixation cross presented in the centre of the screen was calculated for each trial undertaken by each participant. The fixation was determined to be unstable in trials during which the average distance exceeded one degree visual angle.

**Figure 2.**
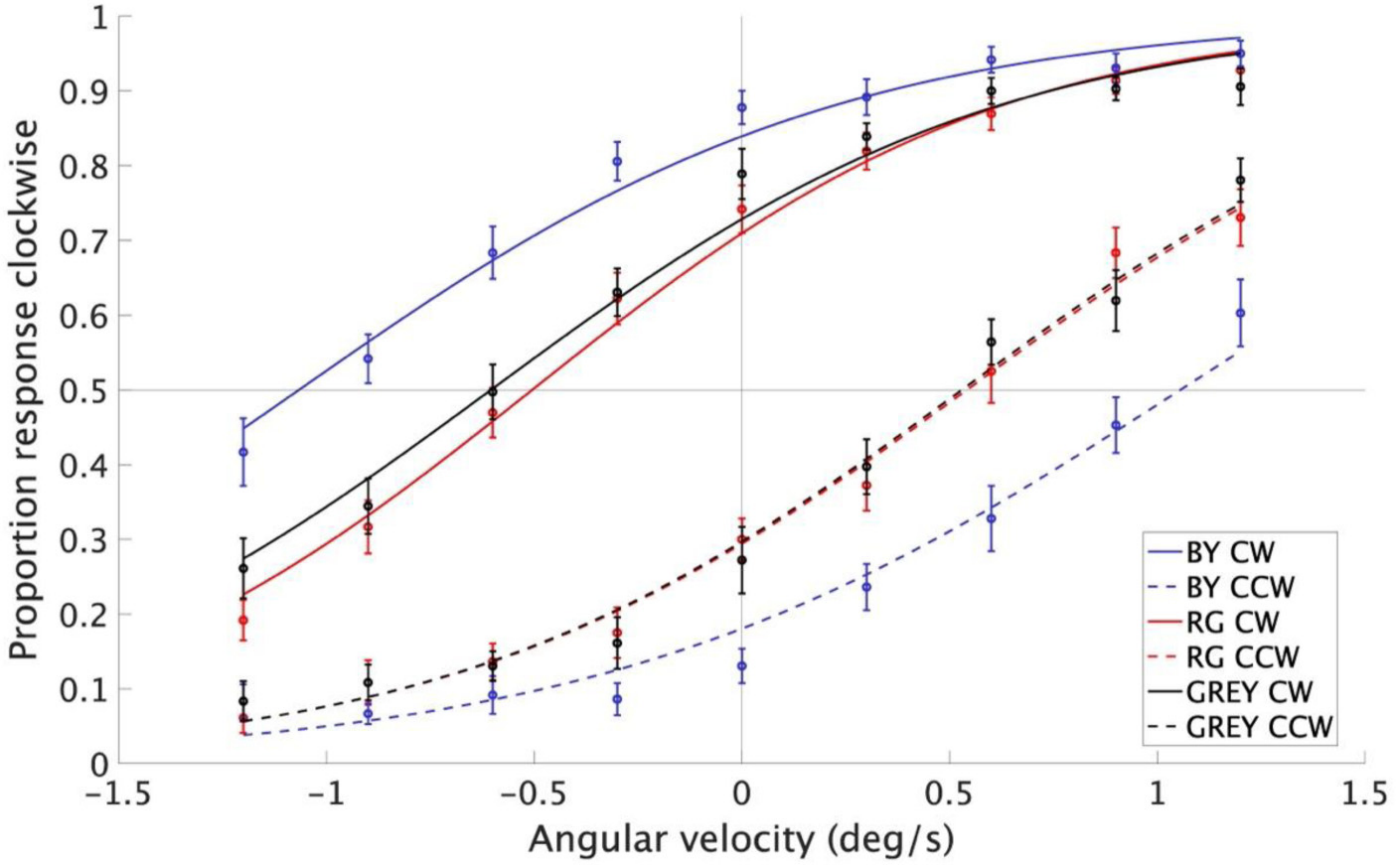
Psychometric curves for the two stimulus types and three colour conditions. The probability of seeing clockwise rotation is plotted against the velocity of physical rotation. The shift of psychometric curve is larger for the BY condition (blue lines) than for RG (red lines) and GREY (black line) conditions. The psychometric curves for RG and GREY are very similar, with those for RG CCW and GREY CCW almost overlapping. Error bars depict ± 1 SEM across participants. Logistic curves fitted to the pooled responses across participants are also plotted.

Figure 2 shows the psychometric curves for the two stimulus types (i.e., CCW and CW) averaged across 20 participants for each colour condition (i.e., BY, RG, and GREY). The probability of seeing CW rotation is plotted against the velocity of physical rotation, where the positive velocity (deg/s) corresponds to the physical rotation in the CW direction. If the participant perceived rotational motion in the stationary CW stimulus, the PSS should be found in the portion of the chart representing negative velocity, and vice versa. This was indeed the case for all three colour conditions as illustrated in Figure 2: the psychometric curves for the CW stimulus type (solid line) are shifted towards negative velocity, whereas those for the CCW stimulus type (dotted line) are shifted towards positive velocity.

**Figure 3.**
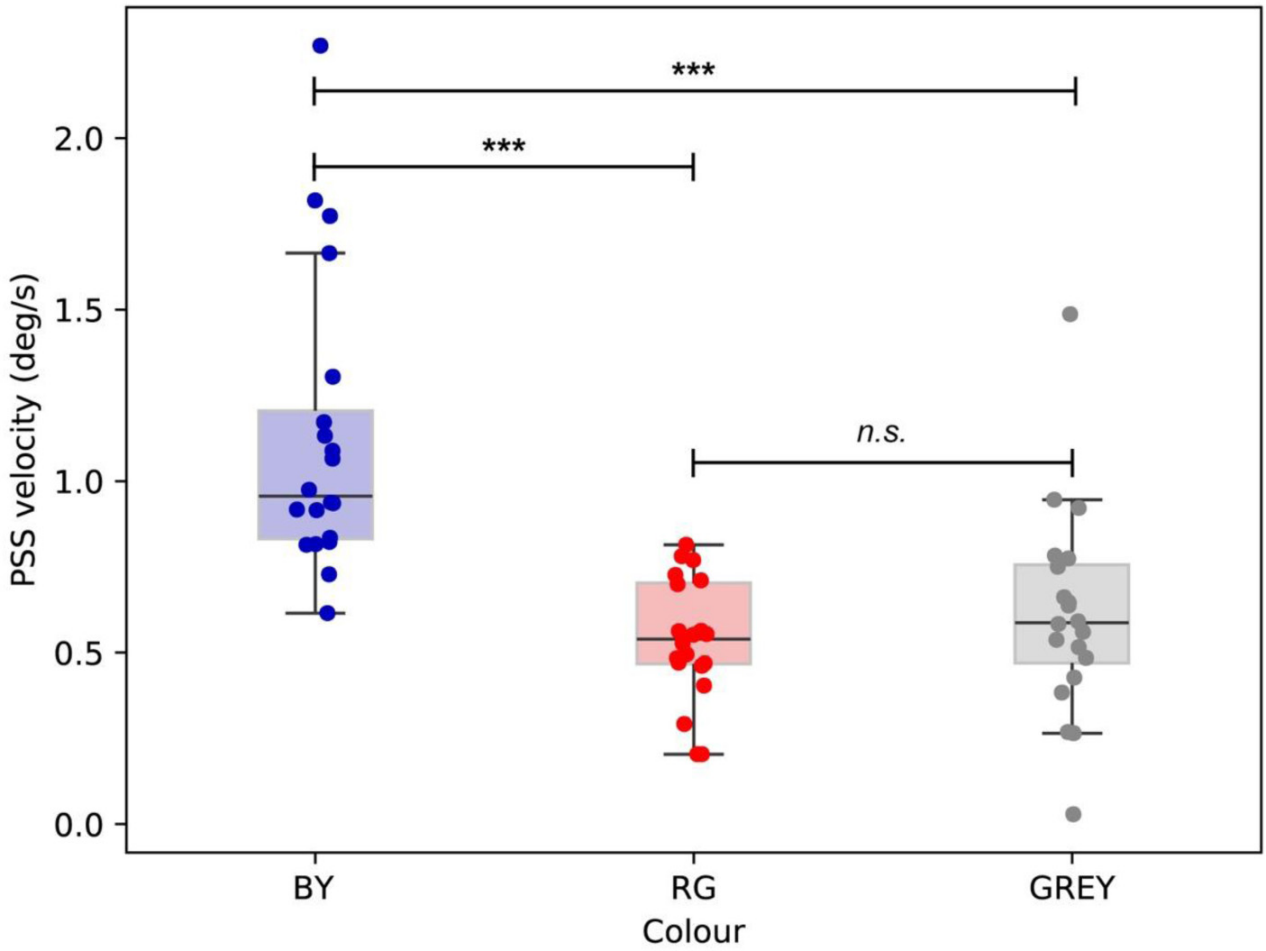
Normalised PSSs for 20 participants for each colour condition. Perceived illusion was greater in the BY condition than in the RG and GREY conditions. *** indicates a significant difference based on the post-hoc analysis (*p* < .001).

Figure 3 shows PSSs for all 20 participants for each colour condition. The box plot highlights that participants perceived greater illusory motion in the BY condition than in the RG and GREY conditions. A two-way ANOVA confirmed the finding: there was a significant main effect of colour (*F*_2, 38_* *= 37.985, *p *< .001, η² = 0.341), but not a main effect of stimulus type (i.e., direction of illusory motion; *F*_1, 19_ = 0.397, *p *= .536, η² = 0.007). There was no significant interaction between colour and stimulus type (*F*_2, 38_ = 1.309, *p *= .282, η² = 0.009). A post-hoc analysis revealed a significant difference between the strength of illusion evoked by the BY stimuli (Holm-corrected *p* < .001) and that evoked by the RG and GREY stimuli. There was no significant difference between the RG and GREY conditions (*p* = .180).

## Discussion

The present study aimed to examine the effect of colours on the perception of the Rotating Snakes illusion. We measured the velocity of physical rotation that cancelled the illusory motion in the opposite direction elicited by the stimulus, which quantified the magnitude of illusion. Results showed that the cancellation velocity was greater for the BY stimuli than for the RG and GREY stimuli. This provides quantitative evidence that supports the observation that the Rotating Snakes illusion is more salient in its BY variation.

Although speculative, there are three possible explanations as to why the BY combination might have contributed to eliciting stronger illusion. First, our findings may be explained by differences in the temporal response feature of the photoreceptors. It is possible that the sluggish response of the short-wavelength sensitive cones compared with the long- and middle-wavelength sensitive cones lead to the stronger perception of illusory motion, because of the larger difference in the transmission latency between blue and yellow than between red and green and between different shades of grey (Baudin et al., 2019). The second interpretation is that the differential recruitment of magno-, parvo-, and koniocellular pathways resulted in the variation in illusory strength. The processing of stimuli comprising blue and yellow would have recruited the koniocellular pathway in addition to the magno- and parvocellular pathways, unlike those comprising red and green, or those in greyscale. While the contribution of the koniocellular pathway in colour processing is established, it is also feasible that the pathway also plays a role in motion-processing (Morand et al., 2000). Not only are some koniocellular cells direction-selective but also MT+ has been shown to receive substantial koniocellular input (Wandell et al., 1999). Furthermore, several subpopulations of koniocellular cells project directly to motion-sensitive areas including MT+, bypassing V1, which makes this pathway distinct from the magno- and parvocellular pathways (Buchner et al., 1997; Moore et al., 2001; Sincich et al., 2004; Yukie & Iwai, 1981). Finally, the possibility that the contrast of chromatic saturation gave rise to the difference in the magnitude of illusion should be considered. Even though the chromatic saturation of the colours that composed the stimuli on the monitor was not measured, it is likely that the contrast of chromatic saturation between blue and yellow was larger than that between red and green under the luminance conditions in our experiment. The larger contrast could have contributed to inducing stronger illusion in the BY stimuli compared to the RG stimuli, although the exact mechanism is unknown. These three explanations are not mutually exclusive, and our findings open new avenues for future studies to explore the mechanisms underlying the effect of colour on motion illusions.

It should be noted that, even though the luminance levels of corresponding areas within each micropattern was physically matched across the three colour conditions using a luminance meter, it would not necessarily have guaranteed subjective equiluminance of corresponding elements for each participant. Although unlikely at the highly suprathreshold luminance levels within the stimuli used in this study, individual differences in sensitivities toward the colours used in the stimuli may have influenced the perception of coloured areas in the micropatterns, and therefore illusory motion.

The present study demonstrates for the first time that colour itself modulates the strength of illusion in the Rotating Snakes illusion when the luminance is matched between the corresponding elements within the patterns; specifically, the blue-yellow combination enhances the illusory percept as compared to the red-green combination and greyscale. While much of the literature on the contribution of colour to motion perception has focussed on red-green input to the computation of motion energy (Cropper & Wuerger, 2005), our findings shed new light to the processing of motion in conjunction with colour and postulate investigation into the role of blue-yellow input to motion processing.
